# Efficacy and safety of radiofrequency ablation for primary and secondary hyperparathyroidism: a retrospective study

**DOI:** 10.1038/s41598-023-44204-5

**Published:** 2023-10-07

**Authors:** Xinguang Qiu, Ming Gao, Danhua Zhang, Feihong Ji

**Affiliations:** Department of Thyroid, The First Affiliated Hospital of Zhengzhou University, 50 Jianshe East Road, Zhengzhou, Henan Province China

**Keywords:** Endocrinology, Oncology

## Abstract

There is now growing interest in the use of Ultrasound-guided radiofrequency ablation (RFA) to treat hyperparathyroidism. But the efficacy and limitations of this treatment have not been described in sufficient detail. Assessing and contrasting the effectiveness and safety of RFA in treating primary hyperparathyroidism (PHPT) and secondary hyperparathyroidism (SHPT). This retrospective study included 57 HPT patients (48 for PHPT and 9 for SHPT) who underwent RFA between January 2017 and April 2021. The serum intact parathyroid hormone (iPTH) and calcium, hyperplastic parathyroid volume, volume reduction rate (VRR) before and after RFA, clinical success rate, symptoms, and complications were analyzed and compared. In SHPT group, bone pain (7/9, 77.8%), skin pruritus (4/9, 44.4%), and multiple hyperplastic parathyroid glands (4/9, 44.4%) were more common compared to the PHPT group. After 12 months of follow-up, the serum iPTH, calcium, and the volume of PHPT and SHPT groups had decreased by more than 60%, 10%, and 90%, respectively (*P* < 0.05). In the VRR, 13 glands of SHPT (72.2%) and 42 glands of PHPT (87.5%) had achieved the clinical success. In addition, the preoperative and postoperative serum iPTH were higher in the SHPT group than in the PHPT group (*P* < 0.05). In terms of the serum iPTH and calcium, the PHPT group had substantially higher rates of clinical success, with 42 patients (87.5%) and 46 patients (95.8%) meeting the criteria, respectively compared to 3 patients (33.3%) and 6 patients (66.7%) of SHPT group (*P* < 0.05). After RFA, the clinical symptoms improved in both groups. The overall incidence of complications (hoarseness and postoperative hematoma) of RFA in the two groups was 10.5% (6/57), and hoarseness (3/9, 33.3%) of SHPT group was more common than PHPT group. All the complications were resolved spontaneously within 12 months after symptomatic treatments. In the treatment of PHPT and SHPT, ultrasound-guided RFA is both successful and safe. PHPT patients have better results in restoring normal iPTH by RFA, and have no considerable difference with the SHPT patients in terms of serum calcium, the volume of the ablation area, and the VRR.

## Introduction

The two kinds of hyperparathyroidism that are most frequently encountered are primary hyperparathyroidism (PHPT) and secondary hyperparathyroidism (SHPT)^1^. Hyperplastic parathyroid glands are often responsible for PHPT, an endocrine condition typified by excessive intact parathyroid hormone (iPTH) and hypercalcemia. Common clinical manifestations of PHPT include bone pain, osteoporosis, kidney stones, skin itching, gastrointestinal symptoms, and neuropsychological symptoms^2,3^. One of the most serious side effects of end-stage renal disease is SHPT. It is frequently associated with parathyroid hyperplasia and chronic increases in serum iPTH and calcium. Common clinical manifestations are bone pain, pathological fractures, cardiac arrhythmias, pruritus, and cognitive impairment. Because the end-stage renal disease has been present for a long time, SHPT does not respond well to conventional medical therapy^1,4,5^. As a result, for symptomatic PHPT and SHPT patients who have experienced treatment failure because of medication interactions, resistance, or adverse effects, parathyroidectomy, as a conventional surgical approach, is commonly chosen to normalize serum iPTH and calcium, arrest disease progression, and alleviate clinical symptoms^6^.

However, PHPT patients often suffer from scarring from classic parathyroidectomy, possible postoperative hypocalcemia, recurrent laryngeal nerve (RLN) damage, hypoparathyroidism, as well as continued oral calcium preparations. As a consequence, they refuse to undergo parathyroidectomy resulting in the aggravation of symptoms such as bone pain and osteoporosis^7^. In addition, SHPT patients frequently have serious diseases of their heart, lungs, and kidneys, a greater risk of general anesthesia-related postoperative complications and poor tolerance to parathyroidectomy are common in SHPT individuals who require the surgery^8^. Therefore, patients with PHPT and SHPT need a treatment modality that is less invasive, effective, fast to recover, and aesthetically pleasing. Currently, the surgical effectiveness of benign thyroid nodules treated with minimally invasive procedures like ultrasound-guided (US-guided) radiofrequency ablation (RFA), is equivalent to that of traditional thyroidectomy^9,10^. In recent years, US-guided RFA has also been gradually used to treat PHPT and SHPT^11,12^. Several literature reports have clearly pointed out that RFA, microwave ablation, and other techniques can validly normalize serum iPTH, calcium, and decrease the volume of glands^13–15^. In this study, patients were separated into PHPT and SHPT groups and all were treated with US-guided RFA. Finding out the assessments and contrast of the efficacy and safety after RFA in the two groups were the objectives of this research.

## Materials and methods

### General clinical data

This study involving human participants was reviewed and approved by the Ethics Committee of the First Affiliated Hospital of Zhengzhou University. It was performed in accordance with relevant guidelines and regulations. Informed consent was obtained from all participants and their legal guardians. The research involving human research participants has been performed in accordance with the Declaration of Helsinki. Our study was a retrospective review of 57 HPT patients (48 for PHPT and 9 for SHPT) who consented to undergo RFA from January 2017 to April 2021. The baseline clinical characteristics were analyzed (Table 1). All patients gave informed permission before the RFA.

**Table 1:** Comparison of baseline demographic and clinical characteristics of the PHPT and SHPT groups before RFA.

Characteristic	PHPT n (%)	SHPT n (%)	Total n (%)	*P*
Number	n = 48 (84.2)	n = 9 (15.8)	n = 57	
Mean age (years)	51.94 ± 13.96	56.44 ± 17.03		0.394
< 55	29 (60.4)	5 (55.6)	34 (59.6)	1.000
≥ 55	19 (39.6)	4 (44.4)	23 (40.4)	
Sex				
Male	8 (16.7)	5 (55.6)	13 (22.8)	0.022
Female	40 (83.3)	4 (44.4)	44 (77.2)	
Single /Multiple				
Single	47 (97.9)	5 (55.6)	52 (91.2)	< 0.001
Multiple	1 (2.1)	4 (44.4)	5 (8.8)	
Serum calcium				
Normal	22 (45.8)	3 (33.3)	25 (43.9)	0.717
Sufficiency	26 (54.2)	6 (66.7)	32 (56.1)	
Thyroid function				
Normal	42 (87.5)	7 (77.8)	49 (85.9)	0.421
Hypothyroidism	5 (10.4)	2 (22.2)	7 (12.3)	
Hyperthyroidism	1 (2.1)	0 (0.0)	1 (1.8)	
Laboratory test				
Serum iPTH (pg/mL)	156.30 (120.38–222.25)	1738.00 (729.45–2637.00)		< 0.001
Serum calcium (mmol/L)	2.72 (2.56–2.90)	2.73 (2.47–3.25)		0.693
Symptom				
Bone pain	10 (20.8)	7 (77.8)	17 (29.8)	0.002
Osteoporosis	5 (10.4)	2 (22.2)	7 (12.3)	0.304
Neuropsychiatric symptoms	7 (14.6)	2 (22.2)	9 (15.8)	0.623
Digestive tract symptoms	9 (18.8)	4 (44.4)	13 (22.8)	0.187
Skin pruritus	2 (4.2)	4 (44.4)	6 (10.5)	0.004
Complication				
Fracture	7 (14.6)	2 (22.2)	9 (15.8)	0.623
Kidney stone	9 (18.8)	3 (33.3)	12 (21.1)	0.380
Comorbidity				
Hypertension	13 (27.1)	5 (55.6)	18 (31.6)	0.124
Diabetes	4 (8.3)	2 (22.2)	6 (10.5)	0.237
Coronary heart diseasr	8 (16.7)	2 (22.2)	10 (17.5)	0.650
Thyroid nodule	15 (31.3)	1 (11.1)	16 (28.1)	0.420
Hyperlipidemia	2 (4.2)	0 (0.0)	2 (3.5)	1.000
Number of ablation	n = 49 (73.1)	n = 18 (26.9)	n = 67	
Maximum diameter				
≥ 2 cm	22 (44.9)	7 (38.9)	29 (43.3)	0.660
< 2 cm	27 (55.1)	11 (61.1)	38 (56.7)	
Volume (mm^3^)	933.05 (395.84–1954.07)	1046.15 (490.09–1552.21)		0.682

*PHPT* primary hyperparathyroidism; *SHPT* secondary hyperparathyroidism; *iPTH* intact parathyroid hormone; *Volume* hyperplastic parathyroid volume.

### Patients

Inclusion and exclusion criteria for SHPT and PHPT patients are listed in Supplementary Information 1.

Inclusion criteria: (1) symptomatic PHPT patients; (2) symptomless PHPT patients who have one of the following conditions: (a) higher serum calcium than the normal range; (b) considerably reduced bone mineral density and a higher probability of suffering a fragility fracture; (c) creatinine clearance < 60 mL/min; (3) SHPT patients who have failed pharmacological treatment; (4) HPT patients with one of the following conditions: (a) at least 1 hyperplastic parathyroid gland found to be amenable to RFA after imaging examination (including ultrasound, computed tomography, X-ray examination, or magnetic resonance imaging); (b) unallowed or rejective to have observational regimens or parathyroidectomy; (c) Aspiration biopsy confirmed benign; (5) The ^99m^Tc-MIBI SPECT/CT reveals elevated radioactive concentrations in the early and late stages^16^.

Exclusion criteria: (1) known history of neck operation; (2) accompanying a coagulation problem; (3) patients with bleeding disorders, serious infectious diseases, or pregnancy; (4) patients with severe underlying diseases that are ineffective for drug treatment; (5) patients with imaging proof of parathyroid cancer; (6) < 12 months of follow-up.

### Pre-ablation examination and preparation

Imaging examination (Supplementary Information 2, 3): Using ultrasound system and ^99m^Tc- MIBI SPECT/CT imaging to locate hyperplastic glands and assess surrounding anatomy: 1. Ultrasound imaging criteria: a well-delimited round, fusiform, or long uniformly hypoechoic masses with complete envelope generally, etc^17–20^. 2. ^99m^Tc- MIBI SPECT/CT imaging criteria: (1) In the early phase, there is a concentration of foci with mild to obvious abnormal radioactivity distribution (radioactivity is uniformly distributed in the thyroid). (2) In the delayed phase, there is a concentration of foci with residual or enhanced abnormal radioactivity distribution (regression or mild radioactivity concentration in the thyroid) ^17–20^. Laryngoscopy was used before RFA to check if RLN damage already existed. Cardiopulmonary function assessment: In order to determine if the patient would be able to withstand RFA, preoperative electrocardiogram, X-ray examination, computed tomography, and magnetic resonance imaging of the chest were all conducted.

### RFA procedure

An experienced RFA specialist performed the treatment with routine electrocardiogram monitoring. The patient entirely exposed the neck by lying supinely. After the neck was sterilized, a color ultrasound system (Toshiba Apli0 500 type color ultrasound system) was used to assess again. To assess the glands and blood perfusion, and 2 ml of ultrasound contrast agent (Sonovel, sulfur hexafluoride microbubbles for injection) was injected into the cubital vein. Local infiltration anesthesia was performed using 2% lidocaine dilution. Under the guidance of ultrasound, a puncture needle (22G, 5 mm tip, STARmed, Korea) was used to inject normal saline into the interstitial space outside the thyroid capsule, esophagus, trachea, nerves, and large blood vessels to create an isolation zone of approximately 5 mm in thickness, which made safe margins for major organs to separated the gland to be ablated from the surrounding anatomy^14,15^. A US-guided RFA needle (18G, 5 mm tip, STARmed, Korea) was inserted into the hypoechoic nodules in the hyperplastic parathyroid region, and mobile ablation was performed at a power of 20–40 W. One ablation was performed for approximately 3 min. The RFA was considered complete when hyperechoic microbubbles fully surrounded the nodule. Contrast-enhanced ultrasonography was then performed again to evaluate the microcirculation perfusion of the lesion, and the needle was slowly withdrawn along the cautery needle tract. The entire RFA procedure took approximately 20–25 min. All the hyperplastic parathyroid glands were ablated. During the RFA process, intermittent communication with the patient was used to check for any abnormal sounds or intraoperative pain. Remember to check patients for vital signs after the RFA and observe whether patients have complications such as neck pain, swelling, and dyspnea. Patients were required to be fasting for 4 h, not talk loudly, press their necks for 30 min, and then apply ice-salt packs for 1–2 h to reduce tissue edema and local bleeding.

### Clinical data collection and follow-up

The follow-up included biochemical examination and ultrasonography. The preoperative and each follow-up serum iPTH, calcium, and the volume of hyperplastic parathyroid glands were collected and evaluated. To calculate the volume of the gland, we used the sphere formula: V = π × abc/6, (V: volume; a: maximum diameter; b and c: other 2 vertical diameters). The volume reduction rate (VRR) was defined by using the formula: VRR = (V_before_ − V_after_)/V_before_^16^.

Evaluation of clinical success: The definition was attaining the complete ablation after RFA. However, due to the short follow-up time in this study, we decided that achieving the clinical success of RFA in the serologic parameters was maintaining the normal levels of serum iPTH and calcium during the follow-up period. The normal range of serum iPTH is 15–65 pg/ml, and the normal range of serum calcium is 2.0–2.7 mmol/L. And the clinical success of RFA in the volume referred to the VRR ≥ 75% at postoperative 6 months and the VRR ≥ 95% at postoperative 12 months. Otherwise, the clinical success of RFA was not achieved^11,16,21,22^.

### Statistical analysis

Using SPSS version 26.0 for all statistical analyses. Continuous variables were expressed as mean ± SD for normal distribution or medians (interquartile range) for skewed distribution. Categorical variables were expressed as frequencies. In order to compare the baseline and follow-up data for each patient, paired t-test (for normal distribution) or paired-sample Wilcoxon-signed-rank-sum-tests (for skewed distribution) were used for continuous variables. Between the PHPT and the SPHPT groups, using the *t*-test (for normal distribution data) or Mann–Whitney U test (for skewed data) to compare continuous variables, and the chi-square or Fisher's exact test to analyze categorical variables. All statistical tests were two-sided, and *P* < 0.05 was considered statistically significant unless stated otherwise.

### Ethics approval

The study involving human participants was reviewed and approved by Ethics Committee of the First Affiliated Hospital of Zhengzhou University. The research was performed in accordance with relevant guidelines and regulations. Informed consent was obtained from all participants and their legal guardians. Research involving human research participants have been performed in accordance with the Declaration of Helsinki.

### Consent to participate

The participants provided their written informed consent to participate in this study.

## Results

### Comparison of baseline demographic and clinical characteristics of PHPT and SHPT patients (Table 1)

The 57 HPT patients were divided into the PHPT group (n = 48) and the SHPT group (n = 9). This study included a total of 67 hyperplastic parathyroid glands. More patients in the SHPT group had multiple hyperplastic parathyroid glands than in the PHPT group (*P* < 0.001). The level of serum iPTH in SHPT group was 1738.00 (729.45–2637.00) pg/mL, which was much higher than that in PHPT group of 156.30 (120.38–222.25) pg/mL (*P* < 0.001). In contrast, the two groups did not have notable differences in the preoperative serum calcium and hyperplastic parathyroid volume (*P* = 0.693; *P* = 0.682). For clinical symptoms, the rate of presenting bone pain (7/9, 77.8%) and skin pruritus (4/9, 44.4%) in SHPT group were significantly greater (*P* = 0.002; *P* = 0.004). Additionally, the two groups did not significantly vary from one another in terms of complications, comorbidities, and the maximum diameter of hyperplastic parathyroid glands.

### Comparison of serum iPTH, serum calcium, volume, and VRR in the two groups before and after RFA at their respective follow-up points

#### The serologic parameters (Table 2)

**Table 2:** The serum iPTH and Calcium at each follow-up before and after RFA in the PHPT and SHPT groups.

	PHPT		SHPT	
Follow-up time	iPTH (pg/mL)	Calcium (mmol/L)	iPTH (pg/mL)	Calcium (mmol/L)
Pre-RFA	156.30 (120.38–222.25)	2.72 (2.56–2.90)	1721.91 ± 1083.91	2.73 (2.47–3.25)
Post-RFA (2H)	75.37 (31.48–106.68)***	2.52 (2.36–2.71)***	1417.39 ± 1036.84**	2.63 (2.34–2.72)*
Post-RFA (1D)	37.08 (15.57–64.03)***	2.41 (2.28–2.59)***	1176.68 ± 952.58**	2.39 (2.10–2.60)*
Post-RFA (1 M)	47.55 (28.25–75.50)***	2.38 (2.29–2.46)***	1148.82 ± 1373.18*	2.36 (2.21–2.54)**
Post-RFA (3 M)	51.05 (25.93–60.60)***	2.43 (2.32–2.52)***	990.08 ± 1446.13*	2.31 (2.22–2.52)*
Post-RFA (6 M)	43.65 (26.63–62.93)***	2.39 (2.30–2.48)***	540.63 ± 832.00***	2.33 (2.22–2.64)*
Post-RFA(12 M)	37.909 (25.93–54.93)***	2.39 (2.31–2.49)***	356.53 ± 496.98***	2.34 (2.24–2.73)*

*PHPT* primary hyperparathyroidism; *SHPT* secondary hyperparathyroidism; *iPTH* intact parathyroid hormone; *RFA* radiofrequency ablation; *H* hour; *D* day; *M* month.

*: *P* < 0.05; **: *P* < 0.01; ***: *P* < 0.001 (compared with the value before RFA).

**Figure 1:**
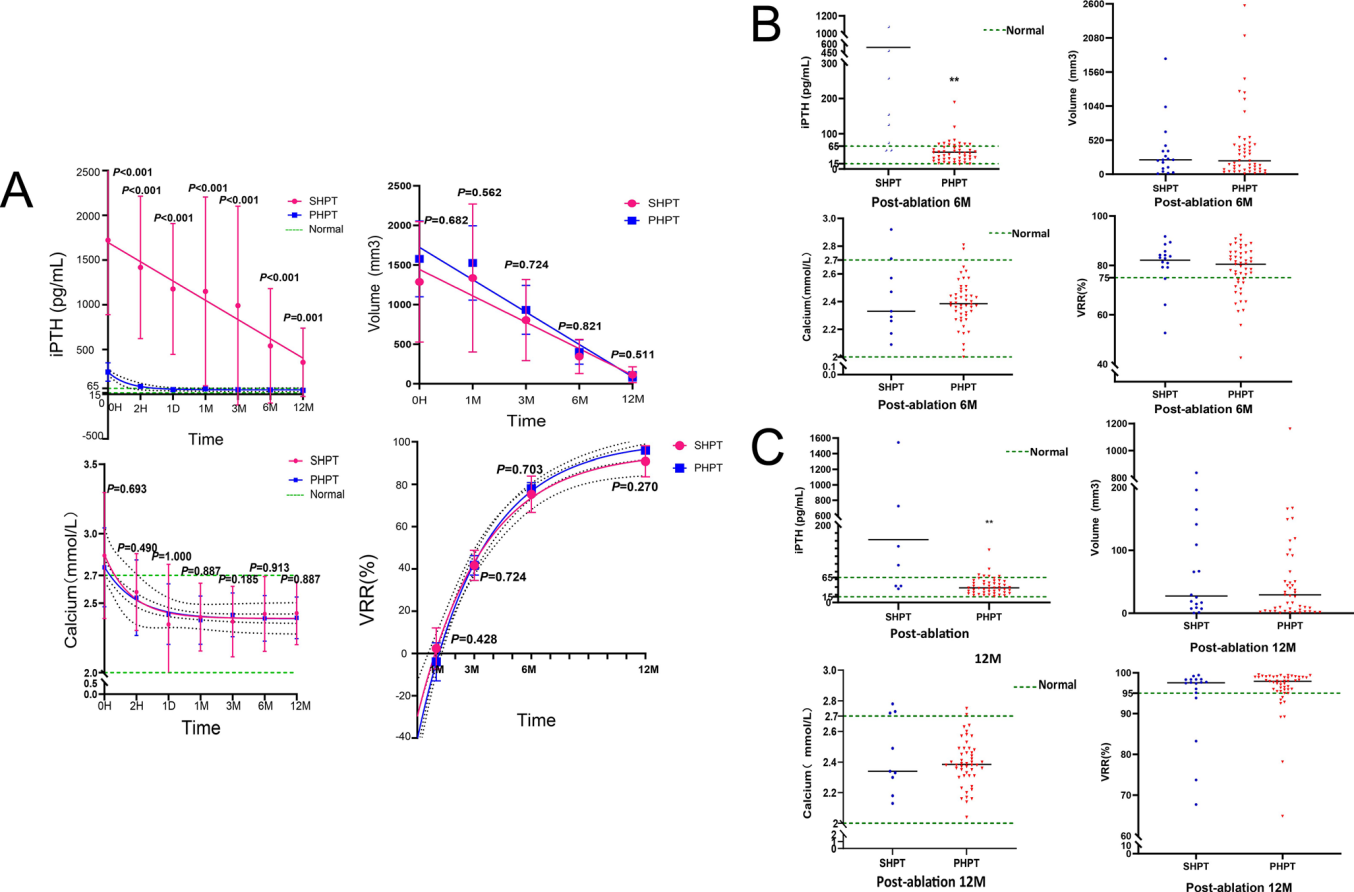
Changes in serum iPTH, Volume, serum Calcium and VRR after RFA for PHPT and SHPT patients. (*Note:* PHPT: primary hyperparathyroidism; SHPT: secondary hyperparathyroidism; Normal: normal range; iPTH: intact parathyroid hormone; Volume: volume of ablation area; VRR: Volume reduction rate VRR = (V_before_ − V_after_)/V_before_; RFA: radiofrequency ablation; 0H: before RFA; H: hour; D: day; M: month; **: *P* < 0.01). (**A**) Changes in serum iPTH, Volume, serum Calcium, and VRR at each follow-up time before and after RFA for PHPT and SHPT patients (*P* < 0.05 at each follow-up time, except the postoperative volume of 1 M for both PHPT and SHPT groups). (**B**) The value of serum iPTH, Volume, serum Calcium, and VRR at 6 M after RFA for PHPT and SHPT groups. (**C**) The value of serum iPTH, Volume, serum Calcium, and VRR at 12 M after RFA for PHPT and SHPT groups.

**Figure 2:**
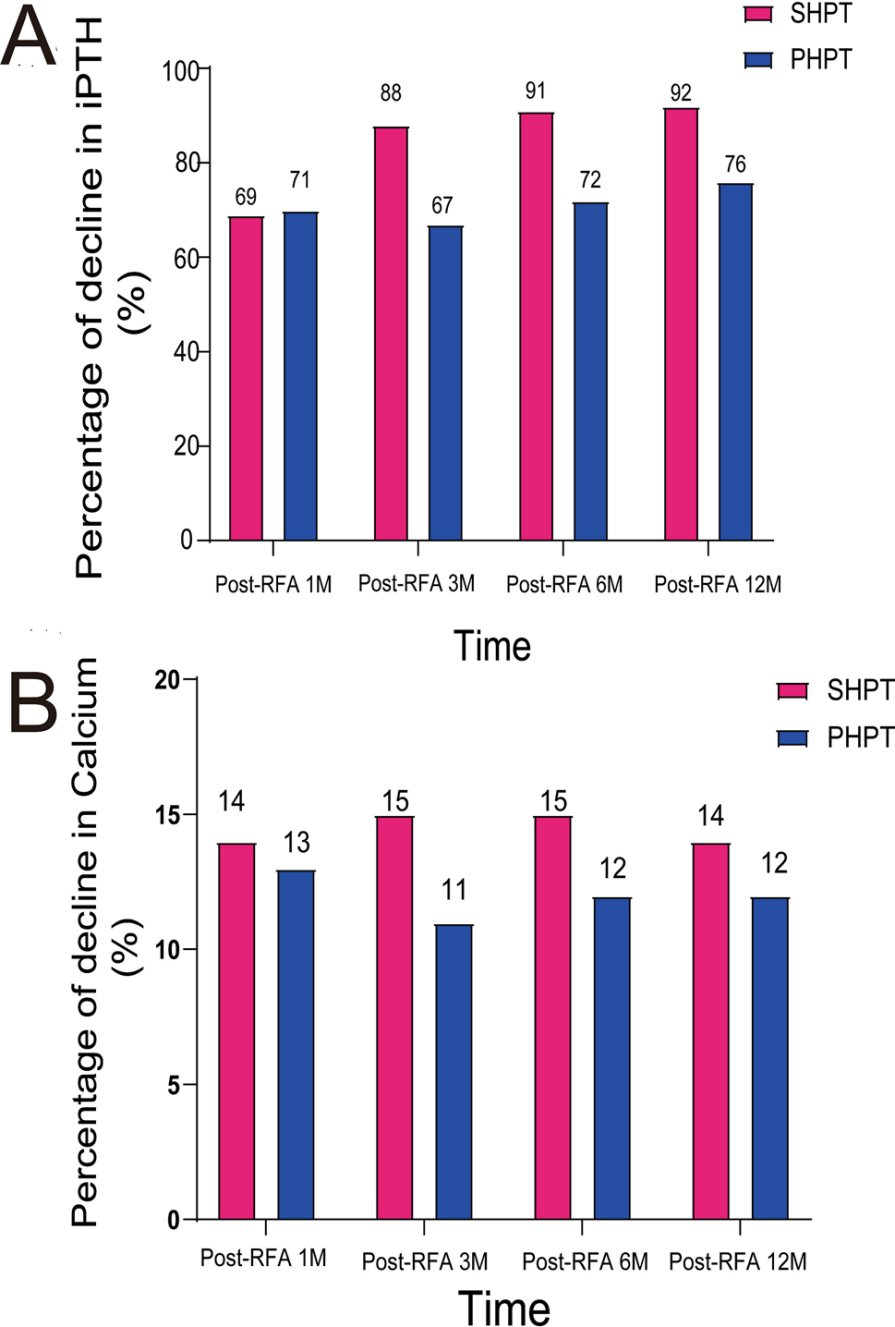
Percentage of decline in PTH and Ca after RFA for PHPT and SHPT patients. (*Note:* PHPT: primary hyperparathyroidism; SHPT: secondary hyperparathyroidism; PTH: parathyroid hormone; RFA:radiofrequency ablation; M: month).

The serum iPTH and calcium of PHPT and SHPT patients at their corresponding follow-up times had significantly decreased (*P* < 0.05), compared with the pre-RFA serum iPTH and calcium. The decrease rates of the serum iPTH and calcium in the two groups at 1 month, 3 months, 6 months, and 12 months after RFA were more than 60% and 10% (Figs. 1A, 2).

For the serum iPTH, in the PHPT group, the serum iPTH of patients at 2 h, 1 day, 1 month, 3 months, 6 months, and 12 months after RFA was 75.37 (31.48–106.68) pg/mL, 37.08 (15.57–64.03) pg/mL, 47.55 (28.25–75.50) pg/mL, 51.05 (25.93–60.60) pg/mL, 43.65 (26.63–62.93) pg/mL, 37.90 (25.93–54.93) pg/mL. Respectively, all of those showed significant decreases compared with the serum iPTH value of 156.30 (120.38 -222.25) pg/mL before RFA (*P* < 0.001, *P* < 0.001, *P* < 0.001, *P* < 0.001, *P* < 0.001, and *P* < 0.001). As for the SHPT group, the serum iPTH of patients at 2 h, 1 day, 1 month, 3 months, 6 months, and 12 months after RFA was 1417.39 ± 1036.84 pg/mL, 1176.68 ± 952.58 pg/mL, 1148.82 ± 1373.18 pg/mL, 990.08 ± 1446.13 pg/mL, 540.63 ± 832.00 pg/mL, 356.53 ± 496.98 pg/mL, respectively compared with the pre-RFA serum iPTH of 1721.91 ± 1083.91 pg/mL, there were significant decreases (*P* = 0.007, *P* = 0.002, *P* = 0.044, *P* = 0.044, *P* < 0.001, and *P* < 0.001).

The serum calcium of PHPT patients at 2 h, 1 day, 1 month, 3 months, 6 months, and 12 months after RFA was 2.52 (2.36–2.71) mmol/L, 2.41 (2.28–2.59) mmol/L, 2.38 (2.29–2.46) mmol/L, 2.43 (2.32–2.52) mmol/L, 2.39 (2.30–2.48) mmol/L and 2.39 (2.31–2.49) mmol/L, respectively compared with the serum calcium before RFA which was 2.72 (2.56–2.90) mmol/L, there were considerable reductions (*P* < 0.001, *P* < 0.001, *P* < 0.001, *P* < 0.001, *P* < 0.001, and *P* < 0.001). For the serum calcium of SHPT patients at 2 h, 1 day, 1 month, 3 months, 6 months, and 12 months after RFA, it was 2.63 (2.34–2.72) mmol/L, 2.39 (2.10–2.60) mmol/L, 2.36 (2.21–2.54) mmol/L, 2.31 (2.22–2.52) mmol/L, 2.33 (2.22–2.64) mmol/L and 2.34 (2.24–2.73) mmol/L, respectively, all of which showed significant decreases compared with the serum calcium value of 2.73 (2.47–3.25) mmol/L before RFA (*P* = 0.021, *P* = 0.011, *P* = 0.008, *P* = 0.011, *P* = 0.013, and *P* = 0.033).

#### The volume of the ablation area and the VRR (Table 3)

**Table 3:** The Volume and the VRR at each follow-up before and after RFA in the PHPT and SHPT groups.

	PHPT		SHPT	
Follow-up time	Volume (mm^3^)	VRR (%)	Volume (mm^3^)	VRR (%)
Pre-RFA	933.05 (395.84–1954.07)		1046.15 (490.09–1552.21)	
Post-RFA (1 M)	989.60 (418.88–1759.10)	3.81 (− 14.57–13.58)	889.84 (404.01–1439.46)	2.37 ± 19.58
Post-RFA (3 M)	529.18 (239.81–997.85)***	44.22 (35.12–50.65)***	604.73 (228.76–885.93)***	41.70 ± 14.32***
Post-RFA (6 M)	203.96 (69.29–451.08)***	80.45 (73.02–85.23)***	219.91 (74.81–373.98)***	75.33 ± 17.25***
Post-RFA (12 M)	29.32 (3.61–96.76)***	97.92 (95.65–98.97)***	27.38 (8.52–147.26)***	90.83 ± 14.58***

*PHPT* primary hyperparathyroidism; *SHPT* secondary hyperparathyroidism; *RFA* radiofrequency ablation; *M* month; *Volume* volume of the ablation area; *VRR* volume reduction rate, VRR = (V_before_ − V_after_)/V_before_.

***: *P* < 0.001 (compared with the value before RFA).

There were notable decreases in the volume of the ablation area of PHPT and SHPT patients at postoperative 3 months, 6 months (Supplementary Information 3), and 12 months, in contrast to the presurgical volumes (*P* < 0.001). What’s more, the VRR at 3 months, 6 months, and 12 months after RFA were much higher than the VRR at 1 month after RFA (*P* < 0.001) (Fig. 1).

The volume of the ablation area of PHPT patients at 3 months, 6 months, and 12 months after RFA was 529.18 (239.81–997.85) mm^3^, 203.96 (69.29–451.08) mm^3^, 29.32 (3.61–96.76) mm^3^. For SHPT patients at 3 months, 6 months, and 12 months after RFA, it was 604.73 (228.76–885.93) mm^3^, 219.91 (74.81–373.98) mm^3^, 27.38 ( 8.52–147.26) mm^3^, which were all significantly smaller than the volume before RFA of 933.05 (395.84–1954.07) mm^3^ and 1046.15 (490.09–1552.21) mm^3^ (*P* < 0.001, *P* < 0.001, *P* < 0.001, *P* < 0.001, *P* < 0.001, and *P* < 0.001). However, the volume of the two groups at 1 month after RFA was 989.60 (418.88–1759.10) mm^3^ and 889.84 (404.01–1439.46) mm^3^, which were not significantly smaller than the preoperative volume (*P* = 0.168, *P* = 0.420).

For the VRR at 3 months, 6 months, and 12 months after RFA, it was 44.22 (35.12–50.65)%, 80.45 (73.02–85.23)%, 97.92 (95.65–98.97)% in the PHPT group, and 41.70 ± 14.32%, 75.33 ± 17.25%, 90.83 ± 14.58% for SHPT patients. All of them were significantly higher than the VRR at 1 month after RFA of 3.81 (− 14.57–13.58)% and 2.37 ± 19.58% (*P* < 0.001, *P* < 0.001, *P* < 0.001, *P* < 0.001, *P* < 0.001, and *P* < 0.001).

### Comparison of serum iPTH, serum calcium, volume, and VRR in the PHPT group with those in the SHPT group

#### The serologic parameters (Table 4)

**Table 4:** The serum iPTH and Calcium at each follow-up before and after RFA in the PHPT and SHPT groups.

	iPTH (pg/mL)		Calcium (mmol/L)	
Follow-up time	PHPT	SHPT	PHPT	SHPT
Pre-RFA	156.30 (120.38–222.25)	1738.00 (729.45–2637.00)***	2.72 (2.56–2.90)	2.73 (2.47–3.25)
Post-RFA (2H)	75.37 (31.48–106.68)	1713.00 (401.65–2332.50)***	2.52 (2.36–2.71)	2.63 (2.34–2.72)
Post-RFA (1D)	37.08 (15.57–64.03)	1436.00 (259.15–2108.00)***	2.41 (2.28–2.59)	2.39 (2.10–2.60)
Post-RFA (1 M)	47.55 (28.25–75.50)	543.80 (205.90–2368.00)***	2.38 (2.29–2.46)	2.36 (2.21–2.54)
Post-RFA (3 M)	51.05 (25.93–60.60)	217.00 (147.20–2017.50)***	2.43 (2.32–2.52)	2.31 (2.22–2.52)
Post-RFA (6 M)	43.65 (26.63–62.93)	155.00 (64.55–782.50)***	2.39 (2.30–2.48)	2.33 (2.22–2.64)
Post-RFA (12 M)	37.90 (25.93–54.93)	146.00 (43.00–540.50)**	2.39 (2.31–2.49)	2.34 (2.24–2.73)

*PHPT* primary hyperparathyroidism; *SHPT* secondary hyperparathyroidism; *iPTH* intact parathyroid hormone; *RFA* radiofrequency ablation; *H* hour; *D* day; *M* month.

*: *P* < 0.05; **: *P* < 0.01; ***: *P* < 0.001 (compared with the SHPT group).

The serum iPTH at pre-operation time, 2 h, 1 day, 1 month, 3 months, 6 months, and 12 months after RFA of the SHPT group were 1738.00 (729.45–2637.00) pg/mL, 1713.00 (401.65–2332.50) pg/mL, 1436.00 (259.15–2108.00) pg/mL, 543.80 (205.90–2368.00) pg/mL, 217.00 (147.20–2017.50) pg/mL, 155.00 (64.55–782.50) pg/mL, 146.00 (43.00–540.50) pg/mL. All of these were substantially higher than those of the PHPT group, which were 156.30 (120.38–222.25) pg/mL, 75.37 (31.48–106.68) pg/mL, 37.08 (15.57–64.03) pg/mL, 47.55 (28.25–75.50) pg/mL, 51.05 (25.93–60.60) pg/mL, 43.65 (26.63–62.93) pg/mL, and 37.90 (25.93–54.93) pg/mL (*P* < 0.001) (Figs. 1, 2). In terms of serum calcium at each follow-up time, the PHPT group had no considerable difference from the SHPT group.

#### The volume of the ablation area and the VRR (Table 5)

**Table 5:** The Volume and the VRR at each follow-up before and after RFA in the PHPT and SHPT groups.

	Volume (mm^3^)		VRR (%)	
Follow-up time	PHPT	SHPT	PHPT	SHPT
Pre-RFA	933.05 (395.84–1954.07)	1046.15 (490.09–1552.21)		
Post-RFA (1 M)	989.60 (418.88–1759.10)	889.84 (404.01–1439.46)	3.81 (− 14.57–13.58)	0.89 (− 5.72–18.50)
Post-RFA (3 M)	529.18 (239.81–997.85)	604.73 (228.76–885.93)	3.81 (− 14.57–13.58)	45.70 (35.93–48.14)
Post-RFA (6 M)	203.96 (69.29–451.08)	219.91 (74.81–373.98)	80.45 (73.02–85.23)	82.09 (71.97–84.57)
Post-RFA(12 M)	203.96 (69.29–451.08)	27.38 (8.2–147.26)	97.92 (95.65–98.97)	97.55 (91.18–98.37)

All *P* > 0.05 (compared with the SHPT group); *PHPT* primary hyperparathyroidism; *SHPT* secondary hyperparathyroidism; *RFA* radiofrequency ablation; *M* month; *Volume* volume of the ablation area; *VRR* volume reduction rate, VRR = (V_before_ − V_after_)/V_before_.

The PHPT group had no considerable difference from the SHPT group in the volume of the ablation area and the VRR at each follow-up time (Fig. 1).

### Comparison of the clinical success of RFA between the two groups

#### Comparison of the clinical success of serum iPTH, calcium, and VRR after RFA between the two groups (Table 6)

**Table 6:** Comparison of the clinical success of RFA between the PHPT group and SHPT group.

Achieved the clinical success at follow-up time	PHPT n (%)	SHPT n (%)	*P*
Serum iPTH			
Post-RFA (6 M)	42 (87.5)	2 (22.2)	< 0.001
Post-RFA (12 M)	42 (87.5)	3 (33.3)	0.002
Serum calcium			
Post-RFA (6 M)	46 (95.8)	7 (77.8)	0.113
Post-RFA (12 M)	46 (95.8)	6 (66.7)	0.024
VRR			
Post-RFA (6 M)	34 (70.8)	13 (72.2)	0.822
Post-RFA (12 M)	42 (87.5)	13 (72.2)	0.281
Improved symptoms	PHPT	SHPT	*P*
Bone pain	10/10	6/7	0.412
Skin pruritus	2/2	3/4	1.000
Neuropsychiatric symptoms	6/7	2/2	1.000
Digestive tract symptoms	8/9	3/4	1.000

*PHPT* primary hyperparathyroidism; *SHPT* secondary hyperparathyroidism; *iPTH* intact parathyroid hormone; *RFA* radiofrequency ablation; *M* month; *VRR* volume reduction rate, VRR = (V_before_-V_after_)/V_before;_ (compared with the SHPT group).

For the serologic parameters, 42 cases (87.5%) in the PHPT group achieved the clinical success in the serum iPTH at 6 and 12 months after RFA, which were significantly more than 2 cases (22.2%) and 3 cases (33.3%) in the SHPT group (*P* < 0.001, *P* = 0.002). Regarding the anticipated serum calcium level, the PHPT group had 46 patients (95.8%) and the SHPT group had 7 patients (77.8%), who achieved the clinical success at 6 months after RFA, with no considerable difference (*P* = 0.113). While the number of SHPT patients dropped to 6 patients (66.7%) at 12 months after RFA, less than the 46 patients (95.8%) in the PHPT group (*P* = 0.024).

**Figure 3:**
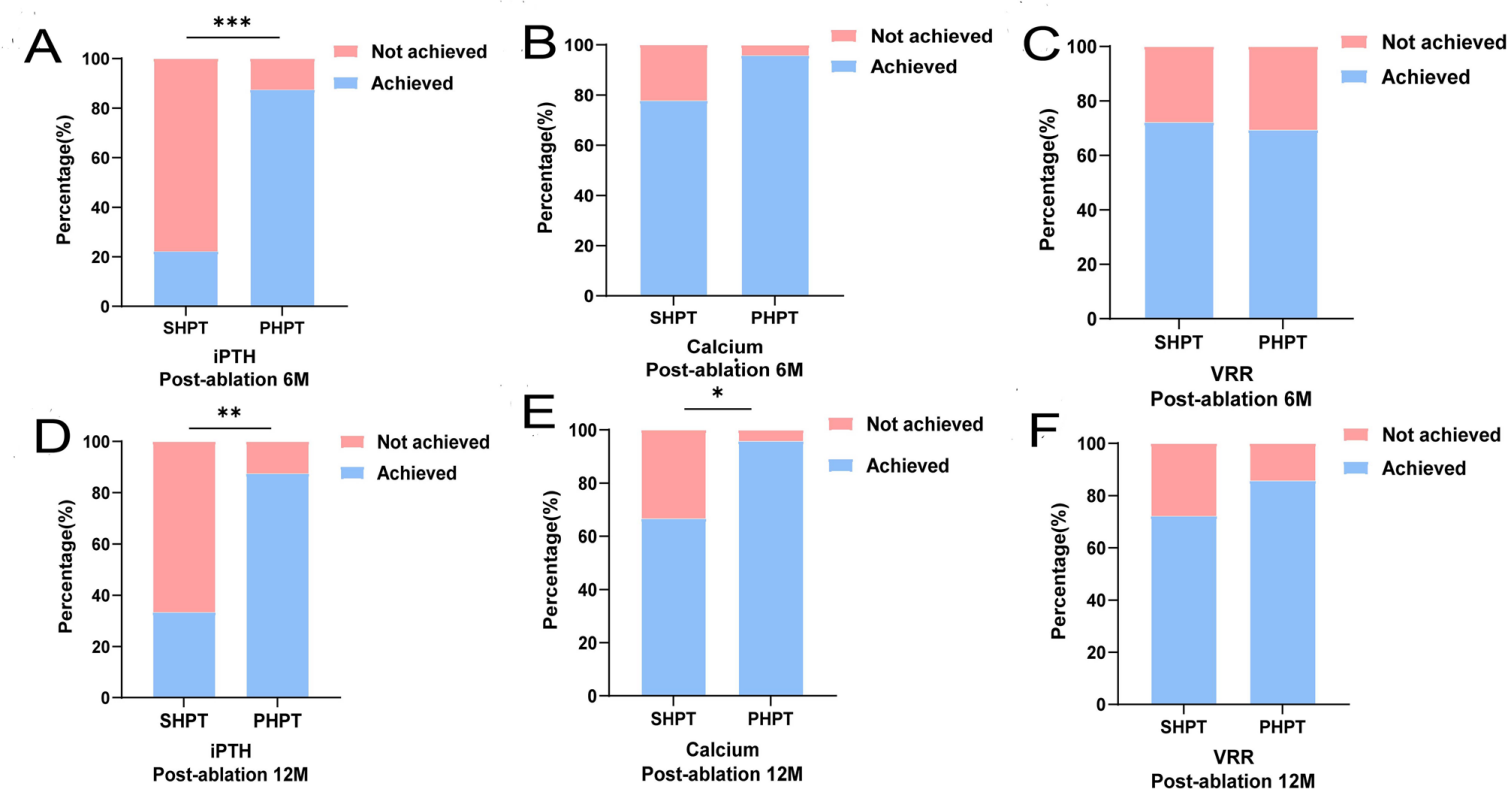
The clinical success rate of serum iPTH (**A**, **D**), serum Calcium (**B**, **E**) and VRR (**C**, **F**) at 6 M and 12 M after RFA for PHPT and SHPT groups. (*Note:* PHPT: primary hyperparathyroidism; SHPT: secondary hyperparathyroidism; iPTH: intact parathyroid hormone; VRR: Volume reduction rate VRR = (V_before_ − V_after_)/V_before_; RFA:radiofrequency ablation; M: month; Achieved: achieve the clinical success; Not achieved: not achieve the clinical success; Percentage (%): the proportion of the number of people who achieved the clinical success for each index in each group at the follow-up time; ***: *P* < 0.001; **: *P* < 0.01; *: *P* < 0.05).

In terms of the VRR at 6 and 12 months after RFA, there were 13 cases (72.2%) in the SHPT group that met the clinical success. This was not discernibly different from the 34 cases (70.8%) and 42 cases (87.5%) in the PHPT group (*P* = 0.822, *P* = 0.281) (Fig. 3).

#### Improvement of clinical symptoms (Table 6)

In the PHPT group, 2 patients (100%) with preoperative skin itching and 7 patients (100%) with bone pain improved after RFA, and the symptoms continued to be relieved until the end of follow-up. The remaining 3 patients who suffered from bone pain also showed relief of symptoms gradually over the follow-up period. Moreover, 6 patients (85.7%) who had neuropsychological symptoms and 8 patients (88.9%) with gastrointestinal symptoms improved after RFA. In the SHPT group, 2 cases (100%) with depression, 6 cases (85.7%) with bone pain, 3 cases (75.0%) with skin pruritus, and 3 cases (75.0%) with anorexia had respectively showed significant improvement after RFA. Clinical symptom relief of RFA was not only satisfying but also not substantially different between the two groups (*P* > 0.05).

### Postoperative adverse reactions (Table 7)

**Table 7:** Complications and Side effects after RFA in PHPT group and SHPT group.

Complications and side effects	PHPT n (%)	SHPT n (%)	*P*
Major complications			
Hoarseness	2 (4.2)	3 (33.3)	0.024
Secondary complication			
Post-operative hematoma	0 (0.0)	1 (11.1)	0.158
Side effects			
Hypocalcemia (numbness of mouth, hands and feet, extremities)	2 (4.2)	2 (22.2)	0.113
Hypoparathyroidism	13 (27.1)	0 (0.0)	0.101
Intraoperative pain	1 (2.1)	1 (11.1)	0.293

*PHPT* primary hyperparathyroidism; *SHPT* secondary hyperparathyroidism.

There were no serious adverse reactions such as dyspnea in the two groups after RFA. Hoarseness suggested that there might be the RLN injury. In the SHPT group, 3 patients (33.3%) developed hoarseness, which was more than the 2 patients (4.2%) in the PHPT group (*P* = 0.024). After giving regular oral methylcobalamin, 1 PHPT patient and 3 SHPT patients recovered within 3 months after RFA, and the other PHPT patient had relief at the end of follow-up by giving extra dexamethasone nebulized inhalation. As for hematoma, only one SHPT patient (11.1%) developed limitedly within 6 h after RFA, which was controlled by temporary pressure with an ice salt bag. The hematoma was completely absorbed at 1 month postoperatively. The two groups respectively had 1 patient who complained of pain during the RFA, and they were relieved after stopping the RFA and having a short rest. 2 PHPT patients (4.2%) and 2 SHPT patients (22.2%) had transient hypocalcemia after RFA. After the administration of oral calcium, the symptoms were resolved within 1 month after RFA. Furthermore, 13 PHPT patients (27.1%) experienced postoperative transient hypoparathyroidism, and they all returned to normal within 3 months without any additional care. The two groups merely had notable differences in the hoarseness of the mentioned adverse reactions.

## Discussion

In recent years, RFA has become a popular alternative to parathyroidectomy for the treatment of PHPT and SHPT patients with apparent symptoms or at risk of affecting other organs, long-term survival, and quality of life due to disease progression. RFA offers the advantages of less trauma, quicker recovery, and lower risk compared to traditional surgical methods^11,12,21,23–26^. However, the efficacy of RFA in treating PHPT and SHPT patients has only been compared in a small number of researches. Therefore, our study aimed to not only compare the changes in four indices (serum iPTH, calcium, ablation area volume, and VRR) before and after RFA in PHPT and SHPT patients but also analyze whether there were any significant differences in the treatment outcomes between the two groups. In our research, we found that RFA could substantially decrease the serum iPTH and calcium levels of patients in the two groups. The volumes of hyperplastic parathyroid glands reduced substantially in both groups, and the median VRR in both groups exceeded 95% at the end of the follow-up period. In addition, 87.5% of PHPT patients and 72.2% of SHPT patients achieved the clinical success in VRR at 12 months after RFA. Previous studies by Ralph P. Tufano et al. also showed that RFA was effective in reducing biochemical parameters (serum iPTH and calcium) and the volume of hyperplastic parathyroid glands to complete disappearance in the PHPT and the SHPT patients, respectively^23,27,28^. That was consistent with the results we obtained indicating that RFA was effective in the treatment of PHPT and SHPT.

Intriguingly, although this study demonstrated that RFA was effective in reducing the serum iPTH values of the SHPT patients. Different from the other three indices, the perioperative, postoperative, and reduced serum iPTH levels of SHPT group were considerably higher than those of PHPT group. What’s more, the percentage of SHPT patients who met the normal serum iPTH (15–65 pg/mL) was also much lower than that of the PHPT group. Similarly, when Li, D et al. studied the minimally invasive treatments of the SHPT patients and the PHPT patients with ethanol ablation, microwave ablation, etc., they found that throughout the brief follow-up time, the serum iPTH of the SHPT patients was greater than that of the PHPT patients and normal levels. But also significantly lower than that of the SHPT patients treated with drug-only intervention. The serum iPTH of the SHPT patients would decrease further after long-term follow-up or secondary ablation^15,29–35^. This research also discovered that the serum iPTH of several SHPT patients increased instead of decreasing after RFA.

The reasons for this result may be: 1. First and foremost, RFA is performed under US-guided rather than direct vision which is unlike parathyroidectomy. In addition, the parathyroid glands are relatively deep, to protect the nearby main blood vessels, RLN, and minimize postoperative complications to the greatest extent, the parathyroid glands may not be completely ablated, resulting in a small number of parathyroid cells remaining. So the serum iPTH values of HPT patients do not return to normal quickly^12,32^. 2. Due to the long-term damage of the kidney and calcium-phosphorus metabolism disorders, multiple parathyroid glands are often stimulated and proliferated in the SHPT patients. While the PHPT patients generally have only one hyperplastic parathyroid gland comparatively^32,36,37^. The SHPT group in our research had significantly more patients with multiple hyperplastic parathyroid glands than the PHPT group. In conclusion, the SHPT patients had more residual parathyroid cells postoperatively. Besides, the poorer body regulation abilities of SHPT patients will cause the disturbance of calcium-phosphorus metabolism to not be completely corrected and continue to stimulate the residual parathyroid cells to increase their proliferation after RFA. We believe that the above-mentioned reasons may result in the serum iPTH of the SHPT group remaining at a higher level than the PHPT group, and appropriately extend the ablation time, increase the ablation power or multiple ablations can be used to achieve the desired outcome^12,32,36,37^. 3. The effect of RFA is continuous. As well as the immediate dying of cancer cells, some scholars believe that the peak of the effect of thermal ablation on cancer cells generally occurs 4–5 days after RFA. So it takes time for parathyroid cells to complete inactivation^38–41^. Whereas in this study, a follow-up time of 12 months might not be sufficient to observe the return of serum iPTH to normal. Therefore, the follow-up time can be appropriately extended in future studies, making it more conducive to exploring whether the serum iPTH will return to the normal level and the long-term efficacy of RFA in the SHPT patients. As for the iPTH rebound phenomenon, some studies pointed out that the body makes adaptive compensatory changes to restore normal calcium homeostasis after RFA. Such as the reduction of ionized calcium activates calcium-sensing receptors on parathyroid cells, which induces PTH secretion and release, or the body develops peripheral PTH resistance^42,43^. For this phenomenon, we require a longer time and more patients to explore and clarify.

However, the two groups had similarities in the values of the last three metrics from the preoperative time to the end of the follow-up of this study. For serum calcium, 46 PHPT patients (95.8%), much more than 6 SHPT patients (66.7%), had achieved the clinical success at 12 months postoperatively. And two other SHPT patients got normal serum calcium after 15 months. We believe that the immoderate serum calcium value is transient, dynamic, and transsexual. It is caused by the body's long-term disease state, poor regulation ability, and oral calcium. Overall, our study on serum calcium is comparable to the earlier research^11^. In terms of the volume of the ablation area, this study found that the index in the two groups observably dropped from 3 months postoperatively and the VRR increased greatly over time. The volume of the ablation area at 1 month after RFA did not decrease significantly compared with that before RFA. Because the scope of RFA was generally extended to the edge of the gland 0.5–1 cm (an isolation zone of approximately 5 mm in thickness formed by normal saline to make safe margins for major organs), for the purpose of cutting off the peripheral blood supply and preventing recrudescence of HPT after RFA. The VRR of the PHPT group could reach 97.92 (95.65–98.97) % at the end of follow-up time and was similar to that of the SHPT group at 90.83 ± 14.58%. Xinyang Li’s research indicated that the VRR at 12 months after RFA of the PHPT patients was 86%^11^. In the studies of Wei, Y et al., the VRR of the HPT patients at 12 months after RFA was 94.6–100%, whereas the ablation area of HPT patients who underwent microwave ablation was completely absorbed at 12 months after RFA^12,28,31,37^. In our study, the decrease in parathyroid volume was slightly different from these prior studies. Probably due to the different ablation methods, different patient conditions, different surgeons, different ablation times, and different power. In future studies, screening patients more carefully, adding the different ablation methods, and extending follow-up times can be used in the more intuitive comparison for which is more effective.

By the end of the follow-up, patients in the PHPT group and the SHPT groups showed great improvement in bone pain, skin pruritus, and digestive tract symptoms. The major complication of this study was hoarseness, for the incidence of the PHPT group was 4.2%, lower than the 33.3% of the SHPT group. The incidences were close to the early published rates of 6–26.671% for the HPT patients treated with ablation^23,32,37^. Multiple thermal stimulation to RLN during RFA of the multiple hyperplastic parathyroid glands in the SHPT group might lead to the difference between the two groups. In our study, the incidences were higher than those of thyroid ablation (1.5%) and parathyroidectomy (3.9%)^23,44^. But all patients with hoarseness improved within the follow-up period after symptomatic treatment, while the researches about thyroid ablation and parathyroidectomy always have patients with permanent hoarseness. The reasons may be that parathyroid glands have nearer locations to the tracheoesophageal groove where the RLN runs than the thyroid, and the RLN is more sensitive to thermal stimulation than mechanical stimulation. We think that minimizing thermal damage to the RLN through sufficient water separation and shorter RFA time is feasible in the future^27^. Moreover, the patients who developed hypocalcemia, transient hypoparathyroidism, and hematoma after RFA all recovered to normal on their own or with medication within 3 months after RFA. In a word, RFA for PHPT and SHPT is a safe treatment option without serious and permanent complications and adverse reactions.

There were several limitations in this study. First, this study had a limited sample size and a brief period of follow-up, which led to a certain selection bias. This type of study needs larger samples, longer follow-up time, and more relevant indicators to fully assess the long-term efficacy of RFA. Secondly, this study only involved the efficacy of RFA on PHPT and SHPT, lacking the comparison of other surgical methods. After that, we can incorporate microwave ablation, laser ablation, parathyroidectomy, and other surgical methods to have a more complete understanding of the efficacy and security of RFA. Thirdly, the chief surgeons in this study included ultrasonographers and endocrinologists in addition to specialists. The varying abilities and experience of doctors may cause bias in treatment, which can be reduced by including only specialist cases.

## Conclusions

In summary, this study provides evidence that US-guided RFA is a secure and efficient technique to treat patients with PHPT and SHPT. SHPT patients have a more complex preoperative profile. There were better outcomes in terms of iPTH and a lower incidence of postoperative hoarseness for PHPT patients. PHPT and SHPT patients have no considerable difference in terms of serum calcium, the volume of the ablation area, and the VRR. For patients with PHPT or SHPT who are not suitable or refuse parathyroidectomy, RFA may be an alternative.

### Supplementary Information

Supplementary Information.

## Data Availability

The datasets generated during and/or analysed during the current study are available from the corresponding author on reasonable request.
