# Supplementary material for: Efficacy and safety of radiofrequency ablation for primary and secondary hyperparathyroidism: a retrospective study
Source: Sci Rep. 2023 Oct 7;13:16949. doi: 10.1038/s41598-023-44204-5 (PMC10560222; doi:10.1038/s41598-023-44204-5)

Supplementary Information

Article Title: Efficacy and safety of radiofrequency ablation for primary and secondary hyperparathyroidism: a retrospective study

journal name: Scientific Reports

Authors'full names and institutions.

Corresponding author: Dr. Xinguang Qiu*
Email: fccqiuxg@zzu.edu.cn
[1]Institution: Department of thyroid, the First Affiliated Hospital of Zhengzhou University, 1 Jianshe East Road, Henan Province, Zhengzhou, China.
ORCID ID: 0000-0002-3816- 1745
Phone number: 86+ 13803710710

Co-first author:
1. Dr. Ming Gao
Email: zzugaoming@163.com
[1]Institution: Department of thyroid, the First Affiliated Hospital of Zhengzhou University, 1 Jianshe East Road, Henan Province, Zhengzhou, China.
ORCID ID: 0000-0003- 1479-0034
Phone number: 86+ 15939019296

Co-first author:
2. Dr. Danhua zhang
Email: 405538395@qq.com
[1]Institution: Department of thyroid, the First Affiliated Hospital of Zhengzhou University, 1 Jianshe East Road, Henan Province, Zhengzhou, China.
ORCID ID: 0000-0001-6801-7536
Phone number: 86+ 13526478812

Second author: Dr. Feihong Ji
Email: 947833961@qq.com
[1]Institution: Department of thyroid, the First Affiliated Hospital of Zhengzhou University, 1 Jianshe East Road, Henan Province, Zhengzhou, China.
ORCID ID: 0000-0002-3163-6250
Phone number: 86+ 16696139285

Supplementary Information 1: Inclusion and exclusion criteria of the PHPT and SHPT groups.

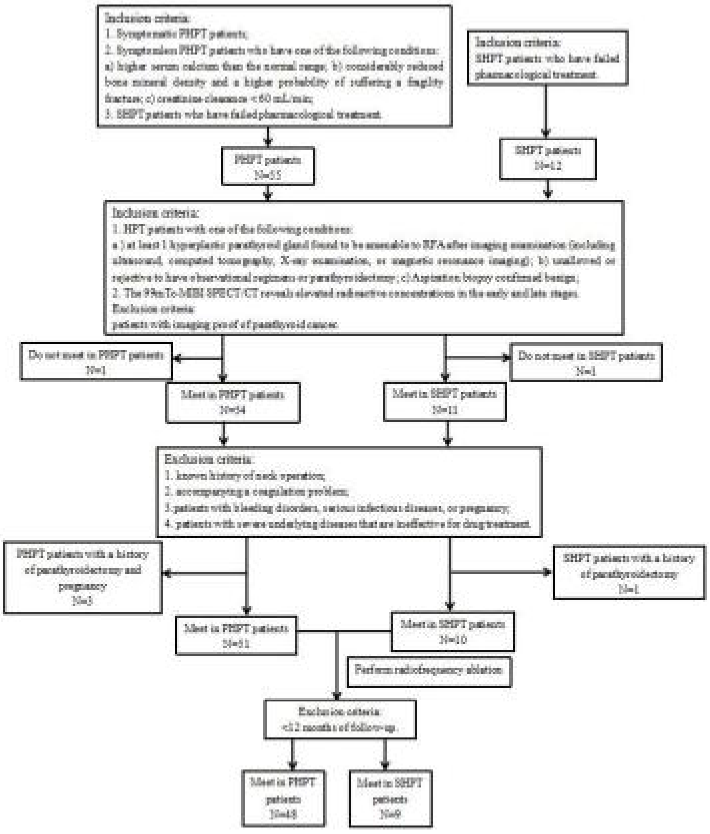

Supplementary Information 2: A 59-year-old male with secondary hyperparathyroidism.99mTc -sestamibi (MIBI) duplex imaging of early (A) and delay (B) phases and SPECT/CT (C) tomography scan showed a hyperplastic right lower parathyroid gland (arrow).

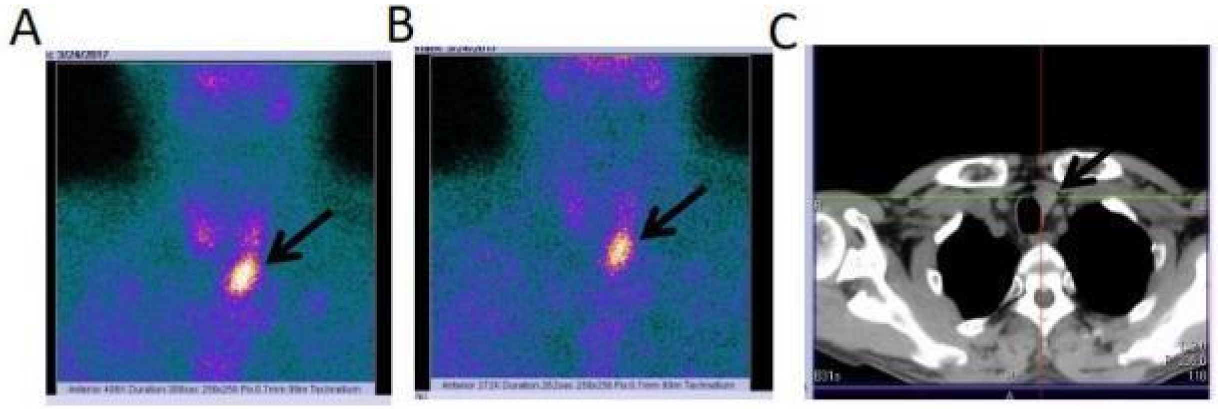

Supplementary Information 3: A 44-year-old female with primary hyperparathyroidism. Ultrasound examination showed a right central posterior hyperplastic parathyroid gland (arrow) before RFA (A) and 6-month after RFA (B). The preoperative size was 23.5mm × 16.5mm ×8mm, and the postoperative size was 13.4mm ×6mm ×3mm. RFA: radiofrequency ablation.

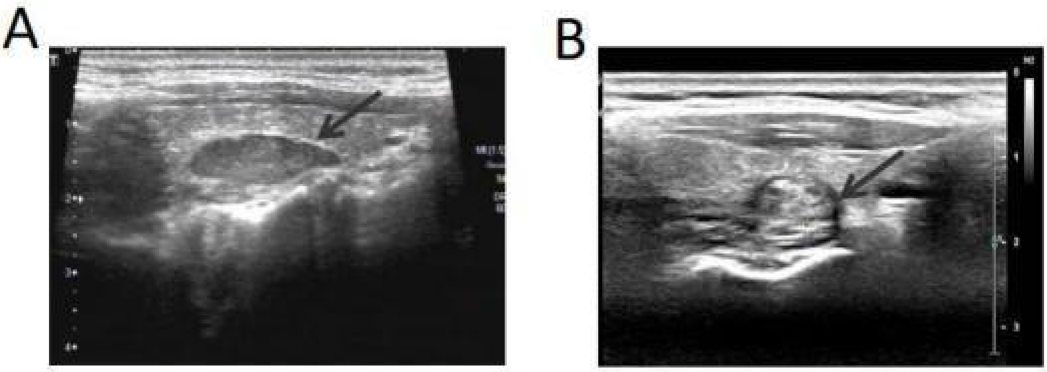

Supplement: Supplementary file 1 — Supplementary Information. [file 41598_2023_44204_MOESM1_ESM.pdf]
